# The Role of Serum Magnesium and Calcium on the Association between Adiponectin Levels and All-Cause Mortality in End-Stage Renal Disease Patients

**DOI:** 10.1371/journal.pone.0052350

**Published:** 2012-12-20

**Authors:** Anastasia Markaki, John Kyriazis, Kostas Stylianou, George A. Fragkiadakis, Kostas Perakis, Andrew N. Margioris, Emmanuel S. Ganotakis, Eugene Daphnis

**Affiliations:** 1 Department of Nutrition and Dietetics, Technological Educational Institute of Crete, Crete, Greece; 2 Department of Nephrology, General Hospital of Chios, Chios, Greece; 3 Department of Nephrology, University Hospital of Heraklion, Heraklion, Crete, Greece; 4 Department of Clinical Chemistry, School of Medicine, University of Crete, Heraklion, Crete, Greece; 5 Department of Internal Medicine, University Hospital of Heraklion, Heraklion, Crete, Greece; University of Florida, United States of America

## Abstract

**Background:**

Adiponectin (ADPN) is the most abundant adipocyte-specific cytokine that plays an important role in energy homeostasis by regulating lipid and glucose metabolism. Studies of the impact of ADPN on clinical outcomes have yielded contradictory results so far. Here, we examined the association of ADPN with serum magnesium (s-Mg) and calcium (s-Ca) levels and explored the possibility whether these two factors could modify the relationship between ADPN and all-cause mortality in patients with end-stage renal disease.

**Methodology/Principal Findings:**

After baseline assessment, 47 hemodialysis and 27 peritoneal dialysis patients were followed- up for a median period of 50 months. S-Mg and s-Ca levels emerged as positive and negative predictors of ADPN levels, respectively. During the follow-up period 18 deaths occurred. There was a significant 4% increased risk for all-cause mortality for each 1-µg/ml increment of ADPN (crude HR, 1.04; 95% CI, 1.01–1.07), even after adjustment for s-Mg and s-Ca levels, dialysis mode, age, albumin and C-reactive protein. Cox analysis stratified by s-Mg levels (below and above the median value of 2.45 mg/dl) and s-Ca levels (below and above the median value of 9.3 mg/dl), revealed ADPN as an independent predictor of total mortality only in the low s-Mg and high s-Ca groups**.** Furthermore, low s-Mg and high s-Ca levels were independently associated with malnutrition, inflammation, arterial stiffening and risk of death.

**Conclusions/Significance:**

The predictive value of ADPN in all-cause mortality in end-stage renal disease patients appears to be critically dependent on s-Mg and s-Ca levels. Conversely, s-Mg and s-Ca may impact on clinical outcomes by directly modifying the ADPN’s bioactivity.

## Introduction

Adipose tissue is not merely a fuel storage organ but an active endocrine organ, producing a variety of bioactive substances termed adipocytokines. Adiponectin (ADPN), the most abundant adipocyte-derived adipocytokine, appears to serve as a central regulatory protein in many of the physiologic pathways controlling lipid and carbohydrate metabolism and to mediate various vascular processes [1]. ADPN displays insulin sensitizing, anti-inflammatory and antiatherogenic properties [2] and it has been associated with better glycemic control, improved lipid profiles and reduced inflammation in diabetic patients [3]. Accordingly, high ADPN concentrations are associated with a favorable cardiovascular risk (CV) profile [4], [5]; however, high ADPN concentrations have also been associated with increased all-cause and CV mortality [6], [7]. Things become more complex when analyzing ADPN concentrations in relation to CV outcomes in chronic kidney disease (CKD) patients. ADPN levels are consistently elevated among patients with CKD and end-stage renal disease (ESRD) [8], [9], being negatively correlated to glomerular filtration rate. However, since ADPN remains elevated after kidney transplantation, other factors in addition to impaired clearance may contribute [10]. Studies in predialysis [11], [12] and hemodialysis (HD) [13], [14], [15] patients showed that low ADPN levels predict worse clinical outcomes. However, more recent and better-powered studies in predialysis [16] and HD [17] patients showed that high, not low, ADPN levels were associated with worse overall and CV mortality. Similarly, high ADPN levels were associated with poor outcomes in patients with coronary artery disease and congestive heart failure [18], [19], [20]. Taken together, while ADPN may be a potential modulator of CV risk, both directly and through the metabolic processes that elevate this risk, epidemiological evidence has not consistently supported elevated levels being protective for adverse outcomes.

The conflicting data concerning the effects of ADPN on outcomes may be caused by differences in study design, inclusion criteria, sex and ethnic background. Also, ADPN’s contradictory role may relate to its concomitant associations with wasting, inflammation, insulin resistance and vascular injuries [21], signifying that differences in mortality may be attributable to differences in the variables that were adjusted for in multivariate analyses. More interestingly, there are factors, such as waist circumference [22] and gender [23], capable of modifying the relationship between ADPN and outcome. In this regard, based on published evidence linking serum magnesium (s-Mg) and calcium (s-Ca) to ADPN in healthy individuals and outcomes in ESRD, we hypothesized that both these two factors could modulate the association between ADPN and outcomes in uremic subjects. Dietary intake of Mg has been associated with increased ADPN levels in the general population [24], [25]. In a recent study [26], both ADPN and intracellular Mg, strongly correlated with each other, were lower in infants with small compared to those with appropriate gestational age, and thus, were both proposed as markers of early fetal growth and insulin resistance in adulthood. Regarding s-Ca, in a population based study, where the associations of s-Ca with cardio-metabolic risk factors were examined, adiponectin had the strongest negative association with corrected s-Ca [27]. Moreover, reduced ADPN levels were detected in patients with primary hyperparathyroidism [28], [29], a state characterized by high s-Ca and insulin resistance. Finally, the adverse impact of low s-Mg [30] and elevated s-Ca [31] or high dialysate calcium (dCa) [32] on clinical outcomes has been well documented in ESRD. Considering all the above, a more thorough examination of the interrelationships of ADPN with s-Mg and s-Ca on all-cause mortality in the ESRD setting is warranted.

The current study was undertaken in ESRD patients to examine a) the existing relationships of ADPN with s-Mg and s-Ca, b) the relationship of ADPN levels with all cause mortality and c) the possible modification effects of s-Mg and s-Ca on the association between ADPN levels and all-cause mortality. Since manipulations of dCa concentrations impact on s-Ca, as they enable alterations on Ca load, the role of dCa itself in relation to ADPN and mortality was also examined.

## Materials and Methods

### Ethics Statement

The study was performed in strict accordance with the ethical guidelines of the Helsinki Declaration and was approved by the Ethical Scientific Committee of the University Hospital of Heraklion, Greece. All study participants provided written informed consent.

### Study Population

The study was performed at the dialysis unit of the University Hospital of Heraklion, Greece. Patients were included when they had been on renal replacement therapy (RRT) for at least 6 months and were 18 years or older. Exclusion criteria included malignant disease, concurrent inflammatory illness and unwillingness to participate. Forty-seven HD and 27 peritoneal dialysis (PD) eligible patients were recruited between October 2007 and November 2008. The etiology of renal failure was hypertensive nephrosclerosis in 20 patients (27%), diabetic nephropathy in 14 (18.9%), glomerulonephritis in 11 (14.9%), interstitial nephritis in 9 (12.2%), polycystic kidney disease in 9 (12.2%) and undetermined in 11 (14.9%). Enrolled HD patients were on standard 4 hours, 3 times weekly dialysis program, using bicarbonate dialysate and high-flux (32%) or low flux (68%) dialysis membranes and aiming for a minimum target KT/V of 1.3. All HD patients were dialyzed against a 0.5 mmol/l Mg dialysate bath, whereas 13 and 34 patients were treated with a low dCa (LdCa) of 1.25 mmol/l and high dCa (HdCa) of 1.75 mmol/l, respectively. PD patients were on a standard continuous ambulatory PD program (4–5 exchanges per day) aiming to a weekly KT/V of 1.7 and creatinine clearance of 70 L/week. All PD patients were treated with a dialysis solution containing Mg at 0.5 mmol/l, whereas 18 and 9 patients were treated with a LdCa of 1.25 mmol/l and HdCa of 1.75 mmol/l, respectively. dCa was selected, aiming at maintaining normal serum calcium and serum parathyroid hormone, after taking into account factors related to Ca load, such as type of phosphorus binder, vitamin D and calcimimetic prescription. In patients prone to intradialytic hypotension, a HdCa of 1.75 mmol/l was frequently used, as a means to improve intradialytic blood pressure instability. dCa prescription remained constant during the follow-up period. Data pertaining to history of CVD, diabetes, arterial hypertension and antihypertensive drugs were retrieved from patients’ medical charts.

### Body Composition

On the day of blood collection, body composition was assessed with bioimpedance analysis (BIA-101; RJL/Akern Systems, Clinton Township, MI, USA). Fat mass and fat free mass were standardized by squared height (m^2^), and expressed in kg/m^2^ as fat mass index (FMI) and fat free mass index, respectively.

### Anthropometric Evaluation

Anthropometric measurements involved body mass index (BMI), waist circumference, triceps skinfold thickness, mid-arm circumference (MAC), mid arm muscle circumference (MAMC) and arm muscle area (AMA). MAMC and AMA were calculated using the formulas:

MAMC (cm) = MAC (cm) –3.14×Triceps Skinfold Thickness (cm).

AMA (cm^2^) = MAMC (cm)^ 2^/12.56.

### Laboratory Measurements

For laboratory testing, blood sample was drawn from a peripheral vein under fasting conditions. Blood samples for determination of interleukin-6 (IL-6), interleukin-8 (IL-8), and ADPN levels were centrifuged, separated and stored at −80°C until analysis. IL-6 and IL-8 were measured with a chemiluminescent immunometric assay, using an Immulite 1000 analyzer (Siemens Medical Solutions Diagnostics Limited, UK). Total human ADPN was measured in serum samples using commercially available enzyme-linked immunosorbent assay kits (R&D System, Minneapolis, MN, USA) according to the manufacturer’s protocol. The lower limit of detection for ADPN was 0.246 µg/ml and the inter- and intra-assay coefficients of variation were 5.8–6.9% and 2.5–4.7%, respectively.

Hemoglobin, serum albumin, prealbumin, transferrin, creatinine, parathormone, total cholesterol, high-density lipoprotein (HDL) and low-density lipoprotein (LDL) cholesterol, triglycerides, Ca and Mg were determined using standard laboratory techniques. C-reactive-protein (CRP) was measured by nephelometry.

### Follow-up

Follow-up data were retrieved from clinical records and/or death certificates by personnel blind to anthropometric, body composition and laboratory assessments. Follow-up began on the date of enrolment and finished upon the death from all causes or 31 December 2011, whichever came first. No patient was lost to follow-up.

### Statistical Analysis

For all statistical analyses, the SPSS/PC 18 statistical package (Chicago, IL) was used. Normally distributed variables were expressed as mean ±SD and non-normally distributed variables were expressed as median (interquartile range). ADPN was examined both as continuous and as dichotomous variable, in the latter case comparing the lowest sex-specific tertile of ADPN (low ADPN group) to the higher (middle and highest) tertiles (high ADPN group). ADPN tertile cut-off points for men were 14.0 and 22.9 µg/ml and 18.0 and 27.3 µg/ml for women, respectively. Differences in baseline characteristics between the groups were tested using the χ^2^ test and the Kruskall-Wallis test as appropriate. Univariate and multivariate regression analyses were used to determine associations between variables. Kaplan-Meier analysis was used to compare survival according to ADPN levels. Cox proportional hazards models were used to evaluate the relationship between ADPN and all-cause mortality initially without adjustment and subsequently adjusting for variables related to ADPN at baseline (s-Mg, s-Ca and dialysis mode) and traditional risk factors (age, albumin, CRP) univariately associated with all-cause mortality at the P<0.05 level. In these models, both Mg and Ca were analyzed as categorical variables. Regarding Mg, patients were classified into two groups based on those who were below the median value (Low Mg group) of s-Mg (2.45 mg/dl) and those above the median value (High Mg group). Regarding Ca, patients were classified into two groups based on those who were below the median value (Low Ca group) of s-Ca (9.3 mg/dl) and those above the median value (High Ca group). Since dCa is the most important prescribed determinant of calcium balance in patients receiving dialysis and, as a consequence, s-Ca is strongly dependent on dCa, as it was in the present study (rho = 0.362; p<0.002), a separate Cox analysis was done by stratifying patients into two groups based on dCa, LdCa and HdCa, as defined previously. Because both these Cox analyses produced almost identical results, we present only the Cox model accounting for s-Ca. Statistical significance was set at the level of P<0.05 (two sides).

## Results

### General Characteristics

The study cohort consisted of 74 patients with a mean age of 65±15 (range 18–83) years. Forty-seven patients (28 men and 19 women, mean age 63±14 years) were undergoing HD treatment and 27 patients (13 men and 14 women, mean age 58±16 years) were treated with PD. Diabetes and CVD were detected in 14 (18.9%) and 15 (20.3%) patients, respectively. There were 45 (60.1%) hypertensive patients and most of them (n = 41) were on antihypertensive drugs [b-blockers, n = 7; calcium channel blockers, n = 19 and angiotensin-converting enzyme inhibitors/angiotensin receptor blockers, n = 25]. RRT was shorter in PD than HD patients (44±37 vs.78±50 months; p<0.05).

### Patients with Low versus High Adiponectin Levels

Twenty five patients had low ADPN levels, as defined in the Methods section, while 49 patients had high ADPN levels. The baseline characteristics of the two groups are shown in Table 1.The two groups did not differ significantly from each other in terms of age, sex and dialysis mode, dCa, RRT vintage, mean arterial pressure, prevalence of diabetes, CVD and hypertension, antihypertensive agent class use and cause of ESRD (data not shown). There was no difference in mean ADPN concentrations between men (n = 41) and women (n = 33) in the study (22±15 vs.25±13 µg/ml), whereas ADPN was lower in HD compared to that in PD patients (21±12 vs.28±16 µg/ml; p<0.05).

**Table 1:** Characteristics of the patients classified into low- and high- adiponectin levels.

Characteristic	Low adiponectin	High adiponectin	P	Adjusted*	
	(n = 25)	(n = 49)		R	P
Epidemiologic and clinical					
Age (yr)	58±13	62±15	0.240	–	–
Sex (males/females %)	56/44	55.1/44.9	0.941	–	–
Diabetes (%)	16	20.4	0.647	–	–
CVD (%)	24	18.4	0.569	–	–
Dialysis mode (HD/PD %)	60/40	65.3/34.7	0.654	–	–
RRT vintage (months)	61±56	68±44	0.556	–	–
Dialysate Ca (Low/High %)	36/49.9	64/53.1	0.369	–	–
Mean arterial pressure (mmHg)	94±14	94±17	0.978	–	–
Hypertension (%)	56	63.3	0.545	–	–
β-Blockers (%)	16	6.1	0.170	–	–
ACEIs +ARBs (%)	44.4	28.6	0.184	–	–
CCB (%)	24	26.5	0.814	–	–
Death from all causes (%)	8	32.7	0.023	–	–
Anthropometric					
Body mass index (Kg/m^2^)	28.1±2.7	24.7±3.1	<0.001	−0.120	0.311
Fat mass index (Kg/m^2^)	10.7±2.5	8.5±3.0	0.003	–	–
Fat-free mass index (Kg/m^2^)	17.4±1.7	16.2±2.2	0.016	−0.120	0.312
Waist circumference (cm)	102.4±9.9	90.9±9.6	0.000	−0.317	0.006
Triceps skinfold thickness (cm	1.8±0.8	1.4±0.7	0.049	0.002	0.984
Mid-arm circumference (cm)	30.9±3.6	27.9±4.1	0.003	−0.232	0.048
Mid-arm muscle circumference (cm)	25.3±3.6	23.4±3.4	0.034	−0.246	0.036
Arm muscle area (cm^2^)	51.9±15.8	44.7±13.2	0.042	−0.238	0.042
Inflammatory					
C-reactive protein (mg/dl)	0.37 (0.3–1.3)	0.46 (0.3–1.2)	0.909	−0.131	0.268
Interleukin-6 (pg/ml)	5.3(3.2–9.0)	5.8 (4.3–11.3)	0.346	0.011	0.926
Interleukin-8 (pg/ml)	10.5 (6.9–19)	13.8 (10–22.4)	0.141	0.266	0.023
Nutritional and biochemical					
Albumin (g/dl)	4.0±0.36	3.85±0.46	0.156	−0.331	0.004
Prealbumin (mg/dl)	30±12	27±9	0.197	−0.109	0.357
Transferrin (mg/dl)	173±41	164±34	0.329	−0.004	0.970
Hemoglobin (g/dl)	11.9±1.2	11.8±1.4	0.883	−0.056	0.643
Creatinine (mg/dl)	8.2±2.7	8.9±2.7	0.924	−0.166	0.159
Total Cholesterol (mg/dl)	174±3.3	171±41	0.407	0.199	0.091
HDL cholesterol (mg/dl)	42±14	50±1 5	0.044	0.207	0.079
LDL cholesterol (mg/dl)	84±25	85±37	0.920	0.251	0.032
Triglycerides (mg/dl)	207 (160–311)	166 (99–216)	0.001	−0.137	0.285
Parathormone (pg/ml)	121 (61–205)	121 (58–169)	0.773	−0.101	0.395
Calcium (mg/dl)	9.3±0.8	9.2±0.8	0.560	−0.293	0.012
Magnesium (mg/dl)	2.4±0.4	2.6±0.5	0.247	0.288	0.014
Phosphorus (mg/dl)	5.2±1.2	5.1±1.1	0.701	−0.076	0.581
Adiponectin (µg/ml)	11.7 (9.7–14.3)	23.5 (20–34.1)	<0.001		–

Values expressed as mean ± SD or median (interquartile range).

CVD, cardiovascular disease; HD, hemodialysis; PD, peritoneal dialysis; RRT, renal replacement therapy; ACEI’s, angiotensin-converting enzyme inhibitors; ARBs, angiotensin receptor blockers; CCB, calcium channel blockers.

* Partial coefficients of correlations between adiponectin and baseline characteristics (anthropometric, inflammatory and nutritional) after correction for fat mass index.

All anthropometric mesurements were lower in the high ADPN group, while no significant differences were found in any of the inflammatory parameters in the two groups. With regard to nutritional parameters, higher HDL cholesterol and lower triglycerides levels were seen in patients with high compared to those with low ADPN.

### Determinants of Serum Adiponectin Levels

Adiponectin adjusted for FMI was inversely associated with almost all anthropometric measurements, serum albumin and s-Ca and positively associated with IL-8, LDL cholesterol and s-Mg (Table 1). In multiple regression analysis, where variables significant in univariate analysis were included, lower BMI, albumin and s-Ca and higher s-Mg and IL-8 were associated with higher ADPN levels (Table 2). HDL cholesterol and sex did not emerge as independent determinants of ADPN levels. This model explained 43% of the variability in adiponectin levels. When dCa was entered instead of serum Ca and FMI instead of BMI, each was significant (data not shown).

**Table 2:** Multiple regression analysis for assessing the predictors of serum adiponectin levels.

Parameter	B	Std error	Std beta	P	Partial r
Constant	97.268	22.221		<0.000	
BMI (Kg/m^2^)	−1.050	0.359	−0.287	0.005	−0.334
Mg (mg/dl)	7.996	3.065	0.260	0.011	0.302
Ca (mg/dl)	−4.914	1.922	−0.270	0.013	−0.296
Interleukin-8 (pg/ml)	0.318	0.136	0.244	0.022	0.273
Albumin (g/dl)	−6.876	3.421	−0.214	0.048	−0.237

Std beta, standardized regression coefficients; r, correlation coefficient;

BMI, body mass index;

s-Mg was inversely correlated with inflammation (IL-6, CRP) markers and arterial stiffness (pulse pressure) and positively with nutritional (transferrin, creatinine) markers, whereas the opposite correlations was observed between s-Ca and these markers (Table 3). Finally, the HdCa group had higher CRP [(0.46 (0.30–1.47) vs. 0.38 (0.30–0.94)] mg/dl; p = 0.040] than the LdCa group.

**Table 3:** Correlations of serum magnesium and calcium with selected baseline parameters.

	Magnesium		Calcium	
Parameter	rho	P	rho	P
Age (ys)	−0.283	0.014	0.194	0.098
Dialysate Ca (LdCa/HdCa)	−0.139	0.236	0.361	0.002
Pulse pressure (mmHg)	−0.274	0.018	0.248	0.033
Transferrin (mg/dl)	0.259	0.026	−0.279	0.016
Creatinine (mg/dl)	0.261	0.021	0.043	0.713
Interleukin-6 (pg/ml)	−0.277	0.017	0.101	0.391
Interleukin-8 (pg/ml)	0.076	0.516	0.232	0.047
CRP (mg/dl)	−0.251	0.031	0.095	0.418

LdCa, low dialysate calcium; HdCa, high dialysate calcium;

CRP, C-reactive protein.

### Adiponectin and Mortality

**Figure 1:**
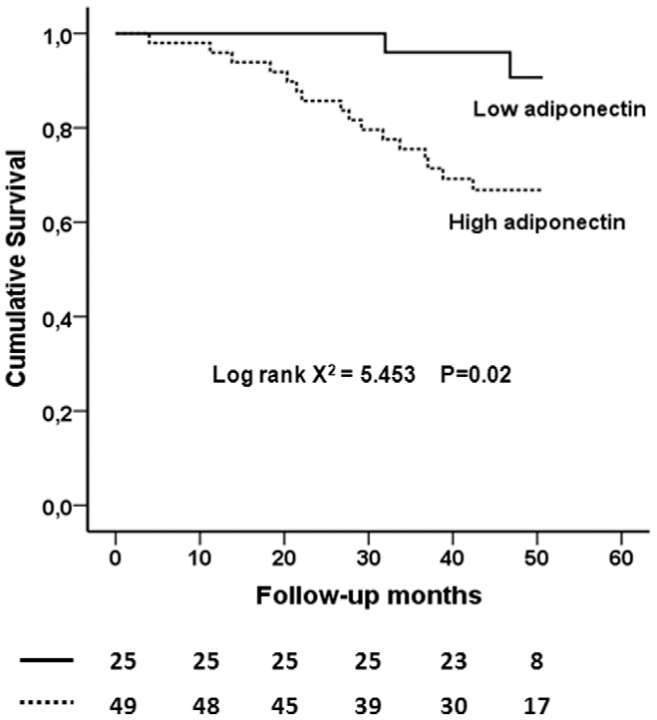
Kaplan-Meier analyses comparing the lowest sex-specific tertile of adiponecting (<14 for men and <18 µg/ml for women) to the higher (middle and highest) tertiles. The number of patients at risk are given below the plot.

During a median follow-up period of 50 months, 18 deaths occurred. Causes of death were CVD (n = 7), infectious complications (n = 6), malignancies (n = 2), intestinal rupture (n = 2) and cirrhosis (n = 1). Patients who died had higher ADPN compared with surviving patients (29±16 vs. 22±12 µg/ml; p = 0.040). Kaplan-Meier analysis (Figure 1) showed that patients in the high ADPN group had a shorter survival rate compared to those in the low ADPN group (67% vs. 92%; p = 0.020). In unadjusted Cox regression analysis (Table 4), every 1 µg/ml of increase in serum ADPN concentration increased the all-cause mortality risk by 4% (crude HR, 1.04; 95% CI, 1.01–1.07). This elevated risk persisted even after adjustment for potential mediators and confounders (HR, 1.07; 95% CI, 1.02–1.12). Results remained similar when dCa (LdCa/HdCa) replaced s-Ca (Low/High Ca groups) in the model; the same was true when BMI replaced albumin, as an index of wasting (data not shown). It is worth mentioning that in the final model, high s-Ca levels and/or HdCa emerged as independent predictors of all-cause mortality, whereas the significant inverse association detected between s-Mg levels and all-cause mortality was lost only after adjustment for age.

**Table 4:** Crude and adjusted hazard ratios of serum adiponectin (per 1 µg/ml) for prediction of all-cause mortality in 74 prevalent ESRD patients.

VariableUnints of increase)	Model 1 (unadjusted)			Model 2		Model 3	
	HR (CI, 95%)		P	HR (CI, 95%)	P	HR (CI, 95%)	P
Adiponectin (1 µg/ml)		1.04 (1.01–1.07)	0.013	1.08 (1.3–1.12)	0.000	1.07 (1.02–1.12)	0.005
Factors related to ADPN							
Low Mg (vs High Mg)				4.04 (1.27–12.8)	0.018	1.16 (0.34–3.96)	0.813
High Ca (vs Low Ca)				5.82 (1.66–20.3)	0.006	5.39 (1.33–21.87)	0.018
PD (vs. HD)				2.93 (1.05–8.19)	0.040	3.14 (0.96–10.31)	0.059
Traditional risk factors							
Albumin (1 g/dl)						0.27 (0.06–1.30)	0.102
CRP (1 mg/dl)						1.51 (0.99–2.30)	0.056
Age (1 yr)						1.09 (1.03–1.16)	0.005

Data adjustment for variables related to adiponectin (model 2), as well as for traditional risk factors (model 3), did not modify the relationship between adiponectin levels and all-cause mortality.

ADPN, adiponectin; PD, peritoneal dialysis; HD, hemodialysis.

### Serum Mg Levels and Dialysate Ca levels Influence the Relationship between Adiponectin and All-cause Mortality

Next, we examined whether adiponectin interacted with s-Mg levels (Low/High Mg groups) and **s**-Ca (Low/High Ca groups) to modify its association with all-cause mortality. An interaction term between ADPN concentration and s-Mg levels was significant (p = 0.002), after controlling for the main effects, albumin, CRP, and dialysis mode. The same was true (p = 0.001) for the interaction between ADPN and s-Ca. Then, the association between ADPN (per 1 µg/ml) and all-cause mortality by subgroups of s-Mg and s-Ca was examined (Table 5). In the low Mg and high Ca groups, ADPN was a significant predictor of all-cause mortality, even after adjustment for age, CRP and albumin. On the contrary, in the high Mg and low Ca groups, ADPN levels were not predictive of outcome in either crude or the adjusted models**.** Of note, a significant (p = 0.001) interaction between ADPN and dCa (LdCa/HdCa) was also observed; the effect modification of dCa on the ADPN-mortality association in magnitude and direction was similar to that observed with s-Ca (data not shown).

**Table 5:** Association of adiponectin with all-cause mortality stratified by serum Mg and dialysate calcium.

Per 1 µg/ml increase of adiponectin					
	E/P	Unadjusted		Adjusted*	
		HR (CI, 95%)	P	HR (CI, 95%)	P
Stratified by s-Mg					
Low Mg group	12/37	1.08 (1.03–1.13)	0.003	1.09 (1.02–1.17)	0.011
High Mg group	6/37	1.04 (0.99–1.09)	0.114	1.03 (0.98–1.07)	0.273
Stratified by s-Ca					
Low Ca group	6/30	1.02 (0.98–1.07)	0.346	1.05 (0.98–1.13)	0.174
High Ca group	11/38	1.09 (1.03–1.15)	0.001	1.08 (1.01–1.16)	0.022

* adjusted for age, C- reactive protein and albumin.

E/P, events/patients.

## Discussion

The present study showed that both s-Mg and s-Ca are major determinants of ADPN levels in ESRD patients. ADPN was positively associated with s-Mg and negatively with s-Ca. In addition, a strong association was demonstrated between high ADPN levels and all-cause mortality, which persisted after multivariate adjustment for possible confounders. Our main finding was that the predictive value of the effect of ADPN levels on mortality was critically dependent on s-Mg and s-Ca concentrations, since high ADPN levels were not predictive of all-cause mortality in patients having high s-Mg and low s-Ca levels.

In this study, we confirm many of the metabolic associations reported previously with ADPN in non-renal [2], [18], [19] and renal patients [13], [16], [17]. Specifically, in our study, lower BMI, albumin, triglycerides and higher HDL cholesterol were associated with higher ADPN levels. In this regard, our findings are in accord with the literature and support the validity of our dataset. Most importantly, this study documents for first time the existence of strong positive and negative associations of ADPN with s-Mg and s-Ca, respectively, in ESRD patients. These associations were independent of each other and independent of body composition, nutritional and inflammatory status. Thus, our data confirm the results of previously reported associations of ADPN with s-Mg [24], [25], [26] and s-Ca [27], [28], [29] in non- renal populations and further extent these findings in the ESRD population, where s-Mg [30] and s-Ca [31] strongly impact on outcomes. The exact mechanisms underlying these associations are not clear, but the fact that common defects in Mg and Ca metabolism are reportedly [33] related to glucose metabolism, provides a possible explanation for this. Indeed, there is enough evidence to indicate that both hypomagnesemia [34], [35] and hypercalcemia [36], [37] are closely associated with insulin resistance. These findings, in concert with the observation that ADPN levels are decreased in patients with type 2 diabetes and in insulin resistance states may at least partially explain the positive and negative associations of ADPN with s-Mg and s-Ca, respectively.

Our results indicated that high ADPN levels were an independent predictor of total mortality in ESRD patients. There was a significant 7% increased risk for death from any cause for each 1-µg/ml increment of ADPN. In addition, the survival rate was significantly lower in patients in the higher sex-specific tertiles compared to those in the lower tertile of ADPN. These data are consistent with recent studies, where there was a 3% to 10.3% increased risk for all-cause mortality for each 1-µg/ml increment of ADPN in CKD [16] and ESRD patients [17]. Since ADPN is presumed to possess antiatherogenic and cardioprotective properties, the association of high ADPN levels with adverse clinical outcomes may be explained by an increased counter-regulatory secretion of ADPN to mitigate inflammation, malnutrition and to protect against endothelial damage and atherogenesis. Although, nutritional and inflammatory statuses were independent determinants of ADPN at baseline, they did not affect the ADPN- mortality association in our study. Alternatively, the existence of a state of adiponectin resistance [38] perhaps due to reduced ligand/receptor activities or down regulation of adiponectin receptors or both may trigger a counter-regulatory increase of ADPN secretion in high risk ESRD patients. Another consideration is that a higher adiponectin level may induce protein energy wasting, a condition associated with malnutrition and inflammation [21]. Reportedly, ADPN may increase energy expenditure and induce weight loss through a direct effect on the brain [39], thus, linking increased ADPN levels to increased mortality in patients with ESRD. Conversely, due to the inverse relationship between adiponectin and fat mass or BMI, weight loss increases plasma adiponectin levels [40] and thus, high ADPN levels in ESRD patients may be a marker of wasting processes and poor prognosis. However, in the present study, adjusting for body composition (BMI) did not alter the effect of high ADPN on mortality.

However, there are also studies, carried out in the general [6], [7], CKD [11], [12], [13] and ESRD [14], [15] [41] populations, in which the lowest levels of ADPN had the worst outcome. Discrepancies among studies in ESRD patients might be explained by differences in the populations studied, inclusion criteria, method of dialysis, confounding influences of covariates, different retention of the different ADPN isoforms in kidney disease [42] and post-translational modifications in the ADPN molecule [23]. In the study by Diez et al [41], comprised of 98 HD and 86 PD patients, an inverse relationship between ADPN levels and all-cause and CVD mortality was reported. Beside a shorter mean follow-up period of 31.2 months, the dialysis vintage was 2.5 (1.7–11.5) months in PD and 12.2 (4.8–43) months in HD patients, whereas the corresponding figures in our study were 36 (18–54) and 80 (36–108) months in PD and HD patients, respectively. In addition PD patients had a mean residual renal function of 3.3 (0.5–6.9) ml/min. It cannot be excluded that the beneficial effect s of ADPN during the early period of renal replacement treatment become harmful over time, particularly when the compensatory increase of ADPN is overwhelming. This assumption is further supported by a population-based cohort of 2484 participants [43], aged 50–75 year, where a higher ADPN was associated with an increased risk of CVD mortality in people with prevalent CVD [HR 1.27 (0.98–1.63)] and with reduced risk in people without CVD [HR 0.90 (0.73–1.11)]. In addition, data regarding s-Mg and s-Ca levels and dialysis prescription were not reported. In contrast, the inverse relationship between ADPN levels and CVD events in a cohort of 227 HD patients [13] can be potentially explained by the Mg and Ca dialysate concentrations used in concert with the findings of the present study, a topic which will be discussed later.

In this study, s-Mg levels were directly correlated with nutritional factors and inversely with pulse pressure, a gross estimate of arterial stiffness, inflammatory markers and age. Furthermore, s-Mg levels predicted total mortality, but this association was largely dependent on age. These findings confirm the results of previous studies supporting a link between low s-Mg levels and atherogenesis [44] or arterial calcification [45], malnourishment and increased risk of death in HD patients [46].

Elevated s-Ca levels and treatment with HdCa, both associated with an increased risk of Ca overload, have also been linked with morbidity and mortality [32], [47], [48]. Our data agree with these reports showing that both increased s-Ca levels and the use of HdCa are associated with adverse clinical outcomes. Indeed, elevated s-Ca levels were associated with a more disadvantageous metabolic risk profile, in terms of increased pulse pressure and IL-8 and lower transferrin, while treatment with a HdCa of 1.75 mmol/l was associated with increased CRP. Both increased s-Ca levels and HdCa predicted independently total mortality. Thus, we provide solid evidence suggesting that Mg deficiency and Ca overload may contribute significantly to malnutrition, inflammation, arterial stiffening and increased CVD death in ESRD patients [30], [31], [46], [47], [48].

The most important finding of this study is that the association between ADPN and mortality varied among subgroups of patients stratified by s-Mg and s-Ca (and/or dCa). In contrast to low s-Mg and high s-Ca (and/or HdCa) groups, ADPN levels were not predictive of death in the high s-Mg and low s-Ca (and/or LdCa) groups. We speculated that the presence of ADPN resistance could be more pronounced in the former groups, due to a worse CVD risk profile, as discussed above. Alternatively, ADPN may not directly affect death risk, but may be a marker of other risks. Another possibility is that s-Mg and s-Ca may impact directly on the bioactivity of ADPN isomers in uremia. ADPN circulates in plasma as a low-molecular-weight (LMW) adiponectin (trimer), middle-molecular-weight (MMW) adiponectin (hexamer) and a high-molecular- weight (HMW) adiponectin (multimer). Although HMW is the most abundant isoform in ESRD patients [49], the distribution and role of each isoform in CKD remains largely unknown. However, emerging evidence suggest that LMW isoforms are associated with better clinical outcomes in both non-uremic and uremic populations, compared to the other isoforms. LMW isoforms were associated with lower CVD risk in children with CKD stage 2–4 [42], and as opposed to HMW isoforms, appear to exert a protective role in older adults with previous coronary heart disease [50] and lead to a reduction of liver cancer risk [51]. Most importantly, a recent study clearly demonstrated that the formation of the fully developed complex HMW structure of ADPN is influenced by the presence of Ca [52]. In both human and mice adipocyte cells, the presence of Ca led to a substantial increased formation of HMW adiponectin, with a corresponding decrease in MMW and LMW isomers, whereas the absence of Ca had the opposite result. These data indicate that low s-Ca and/or potentially high s-Mg levels may be associated with increased LMW isoforms and better outcomes, whereas high s-Ca and/or potentially low s-Mg levels may be associated with HMW isoforms and poor prognosis. This intriguing hypothesis needs to be confirmed in future studies.

This study may also have important clinical implications. Indeed, if s-Mg and s-Ca levels prove to be true effect modifiers of the association between ADPN and mortality, then these findings may impact on clinical practice in the management of ESRD patients, through modifications of dialysate prescriptions, particularly with regard to Mg and Ca and lead to improved guidelines for better outcomes in our high-risk patients. The median s-Mg concentration of 2.45 (2.3–2.7) mg/dl, above which a survival benefit was observed in this study, remained within normal range (1.7 to 2.67 mg/dl). Also, in a previous study [46] using the same dialysate Mg concentration of 0.5 mmol/l, survival was significantly higher in patients with a mean s-Mg concentration above 2.77 mg/dl, a value considered indicative of mild hypermagnesemia. It is possible that if higher Mg dialysate levels had been used, the ensuing higher degree of hypermagnesemia could have resulted in an even better outcome. Since dialysate Mg concentration is an important determinant of Mg balance in both HD and PD patients, a higher s-Mg can be achieved by using a higher dialysate Mg concentration (0.75 mmol/l) than the currently used (0.5 mmol/l) in most countries. We have previously reported [53] that after a four-week treatment with a dialysate Mg concentration of 0.75, 0.5 and 0.25 mmol/l, mean s-Mg concentrations were 2.94, 2.57 and 2.21 mg/dl, respectively. Major guidelines do not comment on dialysate Mg concentrations and trials on this topic with morbidity and/or mortality end points are lacking. A recent review [54] of Mg in dialysis patients indicated that a Mg dialysate of 0.75 mmol/l is likely to cause mild hypermagnesemia, whereas s-Mg levels were mostly normal to low when 0.2 and 0.25 mmol/l Mg concentrations were used. Results were inconsistent (normomagnesemia in most studies) with regard to Mg dialysate of 0.5 mmol/l. A higher survival rate was also observed in patients with a s-Ca concentration below the median 9.3 (8.8–9.7) mg/dl and/or using a LdCa of 1.25 mmol/l. Current guidelines [55] recommend the use of a dCa concentration of 1.25 to 1.5 mmol/l in both HD and PD patients. However a recent study [56] showed that the intradialytic Ca mass balance was nearly neutral using a dCa of 1.25 mmol/l, whereas treatment with a dCa of 1.50 mmol/l resulted in gain of Ca during HD. dCa concentrations as high as 1.75 mmol/l should be avoided to prevent calcium overload and the induction of adynamic bone disease. However, most studies [57] showed a positive effect of HdCa on haemodynamic stability during dialysis compared with LdCa concentrations. Taken all these data together, one could speculate that by increasing dialysate Mg concentration up to 0.75 mmol/l and decreasing dCa concentration from 1.75 or 1.50 to 1.25 mmol/l, the increased ADPN levels in uremia would have rather a beneficial effect on outcomes. Unfortunately, dialysate Mg and Ca levels are not reported in the relevant studies. In the study of Zocalli et al [13], where these were reported, the use of a high Mg dialysate of 0.75 mmol/l and a LdCa of 1.25 mmol/l were associated with a 3% CV risk reduction for each 1-µg/ml increase in plasma ADPN levels**.** Thus, we recommend that s-Mg and s-Ca levels should be taken into consideration when assessing the role of ADPN on outcomes in ESRD and the optimal s-Mg and s-Ca levels required for a survival advantage in relation to ADPN be established.

This study has several limitations. First, due to the small number of patients who died, specific mortality risk (i.e. CVD) could not be determined and generalizability of study results might have been compromised. Generalizability might also have been jeopardized by the low percentage of diabetics, low number of comorbid conditions, lack of other ethnic groups and the fact that a single center participated in the study. Nevertheless, this study enabled us to detect a strong ADPN-mortality association in ESRD patients, the magnitude and direction of which were comparable to those previously reported in relevant studies of the same [17] or larger populations [16]. In the study of Ohashi et al [17], with a sample size (n = 75), number of deaths (n = 15) and a threshold for assessing mortality (15 µg/ml) quite similar to ours, the magnitude of association between ADPN and total mortality was comparable to ours (10.3% vs. 7% adjusted risk increment for each 1-µg/ml increase in ADPN). The robustness of this association did not decrease after adjusting for potential confounder and/or mediators in both pooled and subgroup analyses. Second, we measured total ADPN and not its various isoforms, the reason being that the relevant methodology at the time of our measurements was not available. Notwithstanding, since this was generating hypothesis study, further assessment of ADPN isomers will be necessary to elucidate the difference in the effect of each ADPN isomer on clinical outcomes. In this regard, the first step in testing this intriguing hypothesis is to confirm the presumed positive and negative associations of LMW isoforms with s-Mg and s-Ca, respectively and the corresponding opposite associations regarding HMW isoforms in large cross-sectional studies, and b) then prospectively verify the favorable and unfavorable effects of LMW and HMW isoforms, respectively, on outcomes in relation to targeted s-Mg and s-Ca concentration, through appropriate use and manipulation of Mg and Ca concentration in the dialysis bath. Third, the use of a single baseline measurement to predict events several years in the future. However, serum concentrations of adiponectin seem stable during a period of 1 yr, with minimal short-term variation and high degree of reproducibility [58].

In conclusion, we showed that s-Mg and s-Ca levels can modify the effect of ADPN on all-cause mortality, aiding in unraveling the controversy which surround this association in the existing literature. High ADPN was an independent predictor of death risk only in patients with low s-Mg and high s-Ca levels, respectively, conditions highly associated with a worse CVD risk profile and possibly a marked increase in ADPN resistance. Conversely, the better survival rates seen with high s-Mg and low s-Ca may be caused by altered ADPN bioactivities associated with death risk reduction. Future studies are needed to elucidate the exact roles of s-Mg and s-Ca on ADPN bioactivity in relation to clinical outcomes in ESRD.
